# Promoting Health-Related Cardiorespiratory Fitness in Physical Education: The Role of Class Intensity and Habitual Physical Activity

**DOI:** 10.3390/ijerph17186852

**Published:** 2020-09-19

**Authors:** Miguel Peralta, Diana A. Santos, Duarte Henriques-Neto, Gerson Ferrari, Hugo Sarmento, Adilson Marques

**Affiliations:** 1 CIPER, Faculdade de Motricidade Humana, Universidade de Lisboa, 1499-002 Lisbon, Portugal; mperalta@fmh.ulisboa.pt (M.P.); dianasantos@fmh.ulisboa.pt (D.A.S.); duarteneto@campus.ul.pt (D.H.-N.); 2 ISAMB, Faculdade de Medicina, Universidade de Lisboa, 1649-028 Lisbon, Portugal; 3 Laboratorio de Ciencias de la Actividad Física, el Deporte y la Salud, Facultad de Ciencias Médicas, Universidad de Santiago de Chile, 7500618 Santiago, Chile; gerson.demoraes@usach.cl; 4 Research Unit for Sport and Physical Activity, Faculty of Sport Sciences and Physical Education, University of Coimbra, 3040-156 Coimbra, Portugal; hugo.sarmento@uc.pt

**Keywords:** aerobic fitness, children, PACER, school, 20-m shuttle run

## Abstract

Physical education (PE) has the potential to promote health-related fitness, however, its contribution is still not clear. The aim of this study was to assess whether students’ health-related cardiorespiratory fitness (CRF) improved from the beginning to the end of the school year, and to examine the role of PE class intensity and habitual physical activity (PA) in promoting students’ CRF. This observational study employed a longitudinal design. Participants were 212 7th and 8th grade students (105 boys), mean age 12.9 years old, followed during one school year, from September 2017 to June 2018. The Progressive Aerobic Cardiovascular Endurance Run (PACER) was used to assess CRF at baseline and follow-up. PA was measured using accelerometers. PE class intensity was assessed using the System for Observing Fitness Instruction Time. Findings indicated that from the beginning to the end of the school year, a greater percentage of participants were in the CRF healthy fitness zone (73.1% to 79.7%, *p* = 0.022). Among boys, participating in organized sports (B = 4.61, 95% confidence interval [CI]: 0.33, 8.88) and the percentage of PE time being very active (B = 0.90, 95% CI: 0.44, 1.35) were positively associated with the change in PACER laps. Among girls, daily vigorous PA (B = 0.38, 95% CI: 0.15, 0.60) and participating in organized sports (B = 4.10, 95% CI: 0.93, 7.27) were also positively associated with PACER change, while being overweight or obese (B = −5.11, 95% CI: −8.28, −1.93) was negatively associated. In conclusion, PE was demonstrated to have a positive role in the promotion of CRF, especially among boys, while for girls, habitual PA seems to have a greater contribution. Nevertheless, results and conclusions should be considered carefully, taking into account study limitations, such as the non-direct measures of PE class intensity, CRF, and school setting.

## 1. Introduction

Promoting physical activity (PA) and healthy lifestyles has become a priority for public health authorities worldwide [1]. When considering children and adolescents, school has been purposed as an important setting for achieving this priority. Especially through physical education (PE), the school provides an opportunity for youth to be physically active and promote healthy lifestyles [2].

An important health indicator related to PA is cardiorespiratory fitness (CRF). It is estimated that up to half of the CRF is hereditable [3]. Nevertheless, habitual PA is still considered as the primary means of improving fitness [4]. Systematized evidence revealed strong associations between CRF and youth cardiometabolic health, including blood pressure, cholesterol and triglyceride levels, and glucose tolerance [5]. Moreover, CRF in childhood is suggested to track into adulthood, giving a reasonable insight into future health [6].

There are a handful of field tests that allow for the assessment of CRF in the school setting. School and PE classes may play a significant role in both promoting and monitoring children’s and adolescents’ CRF [7,8,9]. However, among upper-middle- and high-income countries, a substantial decline in CRF has been observed since 1981 [10]. Furthermore, evidence on the contribution of PE classes for promoting children’s and adolescents’ CRF is not consistent [11]. A recent systematic review showed that PE classes can promote child and adolescent CRF, and some factors that may explain the inconsistent findings were identified, including class intensity, age, and weight status [11].

In order to better understand the role of PE classes in promoting CRF, more research is warranted. Previous investigations focusing on the promotion of CRF in PE had mostly implemented cross-sectional or intervention designs [11]. Cross-sectional design studies do not allow us to establish the temporality and direction of the associations. On the other hand, intervention designs, by their nature, alter one or more components of PE classes, including content or intensity, which are important for the promotion of CRF. Taking that into account, there is a need for observational studies employing a longitudinal design. These types of studies allow us to investigate the role of regular PE classes (i.e., not altered by an intervention), and to establish the direction of the associations.

Furthermore, precedent studies mainly examined students from a wide range of grades and ages. Students from different ages and grades may have different PE content, as well as different attitudes toward PE, which may affect the role of PE on promoting PA and CRF [12,13]. Focusing on a narrower range of grades is important to have more specific information. In that regard, the contribution of PE classes to improve CRF seems to be clearer among children and younger adolescents, until 12 years old, than onwards [11]. Also, early adolescence evidences a decline in PA [14,15].

From a public health perspective, promoting and monitoring CRF provides a meaningful strategy to monitor and improve the present and future health status [16,17]. Although PE may be an important platform for promoting children’s and adolescents’ CRF, investigations on its role still present inconsistent results. Therefore, the aim of this study was to assess whether students’ health-related CRF improved from the beginning to the end of the school year. Also, the roles of PE class intensity and habitual PA in promoting students’ CRF were examined.

## 2. Materials and Methods

### 2.1. Study Design and Procedures

This was an observational study. It employed a longitudinal design, where 7th and 8th grade (middle school) students from two schools were followed during one school year, from October 2017 to June 2018. Individual data were collected at baseline, September and October 2017, and follow-up, May and June 2018. Data assessed in these periods included sociodemographic characteristics, PA (assessed only at baseline), and physical fitness. Data on PE classes were collected between January and April 2018.

The two participating public schools were located in Sintra, Portugal. Both schools are from urban areas and located in middle-to-low income neighborhoods. These schools serve a population of approximately 800 students each, and have courses for students from the 5th to the 9th grades. School directors were approached by the research team, and authorization to conduct the study was granted. Afterwards, PE teachers were contacted and asked whether they were willing to participate in the study. Then, students, from the teachers who agreed to participate in the study, were recruited by the research team in a PE class, where the aim and overall aspects of the study were explained, and informed consents were distributed. Students who returned the informed consent signed by their legal guardians were eligible to participate in the study. Before initiating this process, the study protocol was approved by the ethics committee of the Faculty of Human Kinetics, University of Lisbon (no. 19/2017) and by the Portuguese National Data Protection Commission (no. 9249/2017).

### 2.2. Participants

**Figure 1:**
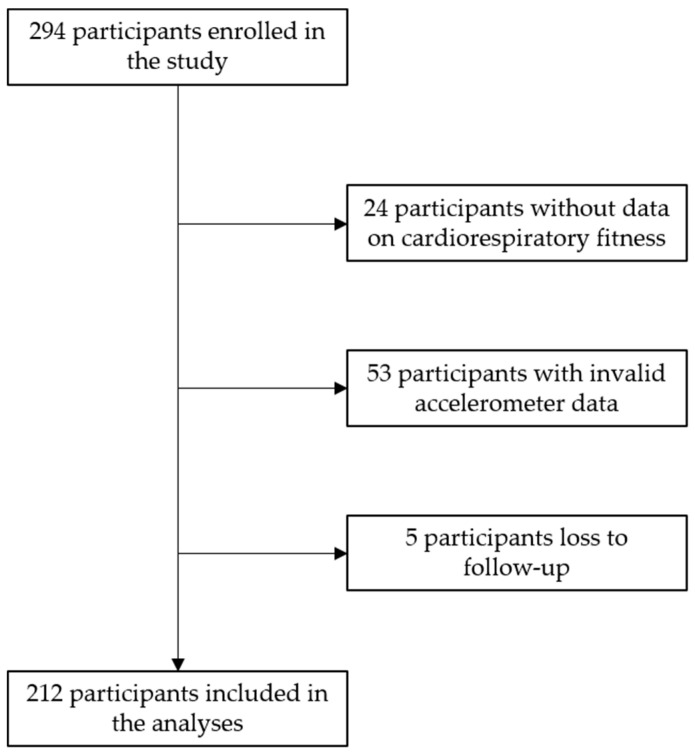
Flow diagram of the study sample.

A total of 294 students, from 21 different classes, enrolled in the study. From the 294 students, five were lost to follow-up (1.7%). Additionally, 24 participants did not have data on CRF, and 53 participants had invalid accelerometer data. Therefore, a total of 212 students (105 boys, 107 girls), mean age 12.9 years old, were included in the analysis. The flow diagram of the study sample is presented in Figure 1.

### 2.3. Measures

#### 2.3.1. Cardiorespiratory Fitness

CRF was assessed using the Progressive Aerobic Cardiovascular Endurance Run (PACER), also known as the 20-m shuttle run, as part of the FITescola battery, which is widely used by PE teachers in Portugal [18]. The main aim of this test was to perform the maximum number of laps with a defined cadence. An audio signal was used to help the participants manage running speed during the test. The test starts with a cadence of 8.5 km per hour and increases progressively 0.5 km per hour every minute. The PACER was performed twice by each participant, at the beginning of the school year (baseline) and at the end of the school year (follow-up). Results from the PACER were recorded in number of laps and VO_2_ peak. (mL/kg/min). For estimating VO_2_ peak, the equations of Saint-Maurice et al. [19] were used, and, afterwards, participants were classified into being in or out of the CRF healthy fitness zone, according to existing standards [20].

#### 2.3.2. Physical Activity

PA was assessed using accelerometers (ActiGraph, GT3X model, Fort Walton Beach, FL, USA), between September and October 2017. The accelerometers used in this research ignored high-frequency vibrations associated with mechanical equipment. All participants were asked to wear the accelerometer on the right side, near the iliac crest, for seven consecutive days, and instructed to remove the devices only when performing water activities (e.g., bathing or swimming) and during sleep. To increase compliance, study staff instructed children how to wear the accelerometer during the initial in-school assessment. Accelerometers were activated on the morning of the first wear day. Individual data were recorded and downloaded into 15 s epochs that were reintegrated into 60 s epochs. This technique is consistent with previously published research [21]. Periods of at least 60 consecutive minutes of zero counts were considered as non-wear time. Only when, in a single day, at least 600 min (10 h) of wear time was recorded, it was considered valid. Participants had to present at least three valid days (including at least one weekend day and two weekdays) in order to have valid accelerometer data.

The cut-points capture the sporadic nature of children’s activity and provide the best classification accuracy among the currently available cut-points for PA in children. Activity levels were expressed in terms of counts per minute, and intensity thresholds were defined according to Evenson et al.’s criteria [22,23]. Accelerometer counts ≥100 counts/min were identified as active time, and posteriorly separated as moderate PA (2296 to 4011 counts/min) and vigorous PA (≥4012 counts/min).

#### 2.3.3. Physical Education Class Intensity

PE class intensity was assessed using the System for Observing Fitness Instruction Time (SOFIT), by a member of the research team. SOFIT is a visual observation instrument previously validated as a measure of PA during PE classes [24]. SOFIT scores activity using a 5-point Likert scale (1—lying down; 2—sitting; 3—standing; 4—walking; 5—very active). A total of 63 PE classes, three PE classes from each student class, were observed. Both schools had three spaces where PE classes were performed (a big outdoors space; a big indoor space; and a small indoor space). One observation was scheduled for each space per student class. For each student class, mean values from the three PE classes were calculated. Intensity values were transformed to percentages of PE time, because class length was not equal for every class. In one school, three PE classes of 50 min were performed per week, while in the other school, two PE classes, one of 50 min, and one of 100 min, were performed per week.

#### 2.3.4. Covariates

Covariates included sex, age, height, weight, body mass index (BMI), age at peak height velocity (PHV), and organized sports participation, at baseline. All participants were weighed to the nearest 0.01 kg on an electronic scale (model 799 SECA, Seca GmbH, Hamburg, Germany) while wearing minimal clothes (t-shirt and shorts) and without shoes. Height was measured to the nearest 0.1 cm with a flexible anthropometric tape on the wall, without shoes. BMI was calculated as body mass (kg)/height^2^ (m). The BMI z-score was calculated, and BMI categories were defined based on the World Health Organization (WHO) classification. Age at PHV was estimated as suggested by Moore et al. [25]. Participants were asked whether they participated in organized sports outside of school (yes/no).

### 2.4. Statistical Analysis

Descriptive statistics were calculated for all variables (means, standard deviation, and percentages) for the entire sample, and separated by sex. Differences between boys and girls were tested by the independent samples t-test, for continuous variables, and chi-square test, for nominal variables. Paired samples t-test and McNemar test were performed to compare the PACER laps, VO_2_ peak, and being, or not, in the healthy fitness zone between the beginning and the end of the school year. Multivariate linear regression models were conducted to assess which factors explained changes in PACER laps from the beginning to the end of the school year, including sex, age, BMI categories, PA (moderate and vigorous), organized sports participation, and percentage of PE time walking and being very active. Models were run for the total sample and by sex, and adjusted to age at peak height velocity, accelerometer wear time, PACER laps at baseline, and each explaining variable. Unstandardized coefficients (B) and standardized coefficients (β) were calculated to have indicators of both measured unit estimates (absolute magnitude) and z-score estimates (relative magnitude). Data analysis was performed using IBM SPSS Statistics version 26.0 (IBM, Armonk, NY, USA). Statistical significance was set at *p* < 0.05.

## 3. Results

Sample baseline characteristics for the entire sample and by sex are presented in Table 1. The majority of participants were normal weight (66.5%), while 22.2% were overweight, and 8.5% obese. Boys spent more time in moderate PA (34.2 min versus 25.9 min, *p* < 0.001) and vigorous PA (22.0 min versus 10.5 min, *p* < 0.001) than girls. Also, a greater percentage of boys than girls participated in organized sports outside of school (55.2% vs. 37.4%, *p* = 0.013). Boys performed more laps in PACER (49.1 versus 30.2, *p* < 0.001), and had a greater estimated VO_2_ peak (48.4 mL/kg/min versus 41.9 mL/kg/min, *p* < 0.001). Most participants, 73.1%, were in the CRF healthy fitness zone; however, a greater percentage of boys was at the CRF healthy fitness zone than girls (83.8% versus 62.6%, *p* = 0.001). On average, participants spent 29.8% of the observed PE time walking, and 24.8% being very active.

Table 2 presents the comparison between aerobic capacity and healthy fitness zone at the beginning and the end of the school year. When considering the total sample from the beginning to the end of the school year, participants improved the number of PACER laps (39.6 to 45.0, *p* < 0.001), and the estimated VO_2_ peak (45.1 mL/kg/min to 46.5 mL/kg/min, *p* < 0.001). Furthermore, the percentage of students in the CRF healthy fitness zone increased (73.1% to 79.7%, *p* = 0.022) from the beginning to the end of the school year.

However, when analyses were stratified by sex, significant changes for all these variables were only found for boys. For girls, differences in estimated VO_2_ peak and percentage in the CRF healthy fitness zone were not significant.

Linear regression models, to explain the change in PACER laps from the beginning to the end of the school year, are presented in Table 3. Considering the entire sample, change in PACER laps was positively associated with being a boy (B = 13.21, 95% CI: 7.64, 18.72), having daily vigorous PA (B = 0.25, 95% CI: 0.10, 0.41), participating in organized sports (B = 4.60, 95% CI: 1.92, 7.28), and the percentage of PE time being very active (B = 0.41, 95% CI: 0.14, 0.68). On the other hand, being overweight or obese (B = −3.01, 95% CI: −5.98, −0.05) was negatively associated with change in PACER laps. It was observed that sex (β = 0.60), followed by daily vigorous PA (β = 0.29), organized sports participation (β = 0.21), and percentage of PE time being very active (β = 0.19) presented the greatest magnitude of association to PACER change.

Interestingly, stratified analysis revealed some sex differences in the variables explaining the change in PACER laps from the beginning to the end of the school year. For boys, participating in organized sports (B = 4.61, 95% CI: 0.33, 8.88) and the percentage of PE time being very active (B = 0.90, 95% CI: 0.44, 1.35) were positively associated with the change in PACER laps. Percentage of PE time being very active had the greatest magnitude of association to PACER change (β = 0.37). For girls, daily vigorous PA (B = 0.38, 95% CI: 0.15, 0.60) and participating in organized sports (B = 4.10, 95% CI: 0.93, 7.27) were also positively associated, while being overweight or obese (B = −5.11, 95% CI: −8.28, −1.93) was negatively associated with PACER change. Minutes of daily vigorous PA had the greatest magnitude of association with PACER change (β = 0.32) (Table 3).

## 4. Discussion

With the aim of examining the promotion of CRF in PE classes, these findings demonstrated that from the beginning to the end of the school year, a greater percentage of participants were in the CRF healthy fitness zone. Also, sex, being overweight or obese, minutes of daily vigorous PA, organized sports participation, and the percentage of PE classes being very active were associated with the change in PACER laps from the beginning to the end of the school year.

Boys are known to have higher CRF than girls from late childhood onwards, and the difference increases throughout the years, reaching approximately 40% in late adolescence [26]. Thus, as expected, in this study, boys performed more PACER laps and had higher estimated VO_2_ peak than girls. Notwithstanding, a greater percentage of boys were in the CRF healthy fitness zone than girls, at both baseline and follow-up. Also, even though findings have demonstrated that from the beginning to the end of the school year a greater percentage of participants were in the CRF healthy fitness zone, sex differences were found. At the beginning of the school year, 83.8% of boys were in the CRF healthy fitness zone, and this percentage increased to 90.5% at the end. On the other hand, 62.6% of girls were in the CRF healthy fitness zone at the beginning of the school year, and 69.2% at the end, however, this difference was not significant. The healthy fitness zone uses health criterion-referenced standards for each sex and age, therefore differences in the percentages of boys and girls in the CRF healthy fitness zone may reveal health disparities. The healthy fitness zone difference between boys and girls is in accordance with previous studies in the Portuguese population [27]. These differences may also be a reflection of the lower levels of PA that girls present when compared to boys [28], which was also observed in this study, as habitual PA is still considered a crucial factor in improving fitness [4].

According to a recent systematic review, age, weight status, and PE class intensity are relevant factors for promoting students’ CRF [11]. In this study, sex, weight status, habitual vigorous PA, participation in organized sports, and the percentage of PE classes being very active were associated with the change in PACER laps from the beginning to the end of the school year. For both boys and girls, age was not associated with changes in CRF, suggesting that both younger and older students can improve. Although this is not in accordance with other studies [11], it should be taken into consideration that 88.7% (*n* = 188) of participants were aged 12 to 14 years old, and thus, age differences may not be identifiable.

Participating in organized sports was associated with a positive change in PACER from the beginning to the end of the school year. Also, sports participation was the only common variable to be associated with changes in PACER in both boys and girls. Previous studies have demonstrated that children and adolescents participating in organized sports have better CRF than their non-participating peers, and that appropriate exercise training is known to increase CRF levels in youth, irrespective of sex, age, or maturity [29,30]. This evidence reinforces that planned and structured exercise programs with adequate frequency, duration, and intensity induce changes in fitness. Considering the school setting, it is possible to plan for activities that fulfil these requirements, especially in PE programs, and therefore promote health-related CRF in PE. However, evidence has demonstrated that most students spend less than 50% of PE time in moderate-to-vigorous PA [31], and that PE time is not associated with students’ CRF [32].

Habitual PA is associated with children’s and adolescents’ fitness [4]. In this study, moderate PA was not associated with a change in PACER, while vigorous PA was only related to a positive CRF change in girls, and when considering the entire sample. Nonetheless, vigorous PA presented the greatest magnitude of association with PACER change among girls. In fact, engaging in regular vigorous PA is most beneficial for improving CRF [33], and is often achieved for those who participated in adequately planned and structured organized sports [29,30]. Surprisingly, in this study, vigorous PA was not associated with PACER change in boys; only in girls. This may be the result of consistent higher levels of PA (moderate and vigorous) in boys, that made it impossible to distinguish levels of vigorous PA between those with positive and negative changes in PACER. Notwithstanding, once again, PE classes have the opportunity to contribute to youth PA levels, including vigorous PA, and thus promote CRF. However, few students spend more than 50% of PE time in moderate-to-vigorous PA [31].

Percentage of PE time walking was not associated with positive changes in CRF. However, percentage of PE time being very active was, although only among boys. Furthermore, among boys, the percentage of PE time being very active was found to have the greatest magnitude of association with PACER change. These are relevant findings for PE, as they demonstrate that PE classes may have an important role in promoting health-related CRF. This is in agreement with a recent systematic review that identified PE class intensity as a key factor for promoting students’ CRF [11]. However, as reported earlier, most of the students spent less than 50% of PE time in moderate-to-vigorous PA [31]. In this study, this was not the case, as more than 50% of PE time was estimated to be in moderate-to-vigorous PA (walking plus being very active). As it is for habitual PA and training, in PE, sufficient intensity together with adequate frequency and duration are key for promoting fitness.

Other aspects of PE besides intensity, such as frequency and duration, can also have an important role in promoting CRF. This study’s participants had 150 weekly minutes of PE. In one school, those minutes were distributed among three classes of 50 min, while in the other school, they were divided into two classes, of 50 and 100 min. Several investigations have proposed that daily PE provides better opportunities for enhancing PA and fitness [34,35,36,37]. Also, the content of PE is of importance. Content that provides greater chances for engaging in moderate-to-vigorous PA (e.g., fitness activities and team games), may help promoting CRF in PE [11]. Therefore, high-quality PE, considering time, frequency, and intensity, can be a successful strategy to promote children’s and adolescents’ CRF levels.

Finally, only for girls, being overweight or obese was negatively associated with the change in PACER laps from the beginning to the end of the school year. A previous longitudinal study showed that although normal-weight girls enrolled in an eight-month PE program improved fitness, their obese peers showed no improvements in response to the same PE program [38]. Overweight and obese youth engage in less habitual PA [39], and are estimated to be 27% and 54% more likely to have school absenteeism, respectively, than their normal-weight peers [40]. These two factors, associated with the fact that PACER is a weight-bearing test, may explain why overweight and obese students have more difficulties in improving CRF.

This study has some limitations that must be acknowledged. The percentage of PE time walking and being very active were estimated through a visual observation instrument (SOFIT), instead of objectively measured (as habitual PA was), which can overestimate PA levels in PE. Nonetheless, SOFIT is validated as a measure of PA intensity during PE classes [24]. Similarly, CRF was assessed using a field test, giving an estimation and not a direct measure [41]. The two schools participating in the study are both from urban areas and middle-to-low-income neighborhoods, which does not take into account participants from other settings. Despite these limitations, the study presents some strengths. Habitual PA was objectively measured using accelerometers. Furthermore, the study employed a longitudinal design, following participants for a whole school year, allowing us to infer temporal directions in the associations assessed.

## 5. Conclusions

From the beginning to the end of the school year, a greater percentage of participants were in the CRF healthy fitness zone; however, differences were only significant for boys. Additionally, being overweight or obese, daily vigorous PA, and participating in organized sports for girls, and organized sports participation and the percentage of PE classes being very active for boys, were associated with the change in PACER laps from the beginning to the end of the school year. Thus, findings suggest that PE has a positive and significant role in the promotion of CRF, especially among boys. Among girls, habitual PA seems to have a greater contribution for the promotion of CRF. From a public health perspective, PE classes can play a significant role in the promotion of health-related fitness, and thus, in improving children’s and adolescents’ health.

## Figures and Tables

**Table 1:** Sample baseline characteristics.

Variables	M (SD) or %			*p*
	Total	Boys	Girls	
	(*n* = 212)	(*n* = 105)	(*n* = 107)	
Age (years)	12.9 (1.0)	13.0 (1.0)	12.8 (1.1)	0.201
Height (m)	1.59 (0.08)	1.61 (0.10)	1.57 (0.07)	0.011
Weight (kg)	52.5 (12.8)	51.6 (12.4)	52.5 (13.2)	0.582
BMI (kg/m^2^)	20.4 (4.1)	19.9 (3.4)	21.0 (4.6)	0.026
BMI (categories)				0.224
Underweight	2.8	2.9	2.8	
Normal weight	66.5	71.4	61.7	
Overweight	22.2	16.2	28.0	
Obese	8.5	9.5	7.5	
Age at PHV (years)	12.9 (0.9)	13.6 (0.5)	12.1 (0.5)	<0.001
Daily MPA (min)	30.0 (11.8)	34.2 (12.5)	25.9 (9.4)	<0.001
Daily VPA (min)	16.2 (12.7)	22.0 (13.6)	10.5 (8.4)	<0.001
Sport participation				0.013
No	53.8	44.8	62.6	
Yes	46.2	55.2	37.4	
PACER (laps)	39.6 (19.4)	49.1 (20.0)	30.2 (13.4)	<0.001
VO_2_ peak (mL/kg/min)	45.1 (6.8)	48.4 (6.9)	41.9 (5.0)	<0.001
CRF healthy fitness zone				0.001
No	26.9	16.2	37.4	
Yes	73.1	83.8	62.6	
% of PE time walking	29.8 (5.3)	30.1 (5.1)	29.5 (5.4)	0.422
% of PE time very active	24.8 (4.8)	25.0 (4.7)	24.5 (4.9)	0.475

Note: M: mean; SD: standard deviation; BMI: body mass index; PHV: peak height velocity; VPA: vigorous physical activity; PACER: Progressive Aerobic Cardiovascular Endurance Run; VO_2_ peak: peak oxygen uptake; CRF: cardiorespiratory fitness; PE: physical education. Differences between sexes were tested by independent samples t-test for continuous variables, and by chi-square test for nominal variables.

**Table 2:** Aerobic capacity and healthy fitness zone at the beginning and at the end of the school year.

Total	M (SD) or %		*p*
	Beginning of The School Year	End of The School Year	
PACER (laps) ^a^	39.6 (19.4)	45.0 (20.7)	<0.001
VO_2_ peak (mL/kg/min) ^a^	45.1 (6.8)	46.5 (7.2)	<0.001
CRF healthy fitness zone (%) ^b^			0.022
No	26.9	20.3	
Yes	73.1	79.7	
			
**Boys**			
PACER (laps) ^a^	49.1 (20.0)	57.3 (20.3)	<0.001
VO_2_ peak (mL/kg/min) ^a^	48.4 (6.9)	50.8 (7.1)	<0.001
CRF healthy fitness zone (%) ^b^			0.039
No	16.2	9.5	
Yes	83.8	90.5	
			
**Girls**			
PACER (laps) ^a^	30.2 (13.4)	32.9 (12.3)	0.007
VO_2_ peak (mL/kg/min) ^a^	41.9 (5.0)	42.3 (4.4)	0.210
CRF healthy fitness zone (%) ^b^			0.210
No	37.4	30.8	
Yes	62.6	69.2	

Note: M: mean; SD: standard deviation; PACER: Progressive Aerobic Cardiovascular Endurance Run; VO_2_ peak: peak oxygen uptake; CRF: cardiorespiratory fitness. ^a^ Tested by paired samples t-test. ^b^ Tested by McNemar.

**Table 3:** Linear regression to explain the change in Progressive Aerobic Cardiovascular Endurance Run (PACER) laps from the beginning to the end of the school year.

Explaining Variables	Change in PACER from The Beginning to The End of The School Year					
	Total		Boys		Girls	
	B (95% CI)	β	B (95% CI)	β	B (95% CI)	β
Sex						
Girl	0.00 (ref)		NA	NA	NA	NA
Boy	**13.21 (7.64, 18.72)**	**0.60**				
Age (years)	0.59 (−0.80, 1.98)	0.06	−1.00 (−3.36, 1.37)	−0.08	0.95 (−0.60, 2.50)	0.10
BMI categories						
Not overweight or obese	0.00 (ref)		0.00 (ref)		0.00 (ref)	
Overweight or obese	**−3.01 (−5.98, −0.05)**	**−0.13**	0.82 (−4.22, 5.85)	0.03	**−5.11 (−8.28, −1.93)**	**−0.25**
Daily MPA (min)	−0.09 (−0.23, 0.06)	−0.09	−0.09 (−0.31, 0.13)	−0.09	−0.10 (−0.30, 0.09)	−0.10
Daily VPA (min)	**0.25 (0.10, 0.41)**	**0.29**	0.16 (−0.05, 0.36)	0.19	**0.38 (0.15, 0.60)**	**0.32**
Sport participation						
No	0.00 (ref)		0.00 (ref)		0.00 (ref)	
Yes	**4.60 (1.92, 7.28)**	**0.21**	**4.61 (0.33, 8.88)**	**0.20**	**4.10 (0.93, 7.27)**	**0.21**
% of PE time walking	−0.11 (−0.36, 0.14)	−0.05	−0.36 (−0.77, 0.05)	−0.16	0.02 (−0.28, 0.33)	0.15
% of PE time very active	**0.41 (0.14, 0.68)**	**0.19**	**0.90 (0.44, 1.35)**	**0.37**	0.02 (−0.29, 0.32)	0.15

Note. PACER: Progressive Aerobic Cardiovascular Endurance Run; CI: confidence interval; BMI: body mass index; MPA: moderate physical activity; VPA: vigorous physical activity; PE: physical education; NA: not applicable. B coefficients show the unstandardized values, and β coefficients show the standardized values. Model was adjusted for age at peak height velocity, accelerometer wear time, PACER laps at baseline, and each presented variable. Significant values are in bold.
